# Implementing a physical healthcare clinic in a CAMHS neurodevelopmental population

**DOI:** 10.1192/bjo.2021.825

**Published:** 2021-06-18

**Authors:** Helen Bruce, Sharika Mansoor, Sacha Evans

**Affiliations:** 1 UCL Division of Psychiatry; 2 Barts Health NHS Trust & East London Foundation Trust; 3 Great Ormond Street Hospital

## Abstract

**Aims:**

To establish a physical health clinic in a community CAMHS to monitor patients in the NDT who are on stimulant/antipsychotic medication

To re-audit to assess adherence to physical health monitoring in accordance with guidelines

**Background:**

Studies have indicated that people with severe mental illness have higher rates of mortality and are prone to development of physical health problems compared to the general population. Monitoring physical health is therefore important as it allows early detection and intervention where appropriate.

**Method:**

17 out of 120 patients in the NDT were identified as taking either an antipsychotic (8 patients) or stimulant medication (9 patients). Physical health data required were determined by local policy and the Maudsley guidelines.

Parents were invited to attend the clinic with their child through telephone calls. Height, weight, blood pressure and pulse were measured in the appointment. A blood test form was provided for parents to take to local outpatient phlebotomy services. A GP letter was sent with the results of the physical health check with a request to conduct an ECG and notify us of any abnormal results. Feedback forms were collected from parents to share their experience of attending the physical health clinic.

Five patients were identified as having difficulty attending the CAMHS clinic due to refusal/challenging behaviour. For three patients, school visits were organised to conduct a physical health check.

**Result:**

The results from the second round of the audit indicate an overall improvement in the adherence to monitoring guidelines for antipsychotic and stimulant medication. This was particularly evident for the patients on antipsychotic medication. Feedback collected from parents regarding the service provided was also positive.

**Conclusion:**

The physical health clinic identified challenges preventing 100% compliance in all patients. This included difficulties with parents bringing their child to CAMHS due to challenging behaviour. In a few of the patients, it was possible to solve this issue by conducting a school visit.

It was also observed that there were multiple instances where challenging behaviour lead to inability to conduct certain tests including blood pressure, blood tests and ECG. Additional strategies should be considered to improve compliance.

A notable issue that also arose from the development of the physical health clinic was that it was unclear how to obtain an ECG at CAMHS.

Continuation of the clinic as well as extension to include patients within other teams at Tower Hamlets CAMHs would be recommended.

